# Assessing the Effect of Board Gender Diversity on CSR Reporting Through Moderating Role of Political Connections in Chinese Listed Firms

**DOI:** 10.3389/fpsyg.2021.796470

**Published:** 2021-12-31

**Authors:** Riffat Shaheen, Hailan Yang, Muhammad Yaseen Bhutto, Hussaini Bala, Fahad Najeeb Khan

**Affiliations:** ^1^Department of Finance, Economics and Management School, Wuhan University, Wuhan, China; ^2^Business School, Shandong Jianzhu University, Jinan, China; ^3^Department of Accounting, Faculty of Administrative Sciences and Economics, Tishk International University, Erbil, Iraq; ^4^Noon Business School, University of Sargodha, Sargodha, Pakistan

**Keywords:** board gender diversity, CSR reporting, political connections, China, corporate governance

## Abstract

This study departs from existing work on board gender diversity (BGD) and corporate social responsibility (CSR) reporting by analyzing and explaining the mechanism by which gender-diverse boards in politically embedded firms (PEFs) affect firms’ CSR reporting choices in a unique institutional setting of Chinese listed firms from 2010 to 2018. The following main results are obtained. First, having female directors and executives with political connections (PCs) on corporate boards improves the CSR reporting of firms. Firms with PCs have a greater possibility to issue CSR reports than their non-connected counterparts. Second, firms that have both gender diversity and PCs on their boards of directors are more likely to engage in CSR reporting. There is an indication that the presence of PCs on boards can strengthen the effect of female directors on firms’ CSR reporting. Third, the presence of female directors on corporate boards has a stronger relationship with CSR reporting in PEFs than in non-PEFs. The study concludes that both BGD and PCs on corporate boards positively influence the diffusion of CSR-related practices in the Chinese business environment.

## Introduction

Corporate social responsibility (CSR) – previously viewed as a voluntary activity undertaken by businesses to improve social and environmental conditions – has evolved into a strategic issue on the agendas of boards of directors (BODs) and has been planned and discussed at the top management level to fulfill the business’ social responsibility (Mackey et al., 2007; Calderón et al., 2020). Despite its global reach, CSR remains contextual both in terms of its corporate and national environments (Moon, 2004), and its importance varies over time and across countries. It has been a well-recognized concept in developed countries for decades, but it is a relatively recent addition to the political and business agendas of emerging countries. China is a unique case in emerging economies in terms of CSR due to its rapid growth, which has resulted in severe damage to the country’s environment and society (Lin and Ho, 2011), and the country has long been accused on both national and global scale of being negligent to environmental and social problems. Since 2004, the Chinese government has introduced and enforced numerous CSR reforms to contribute to sustainable economic development, and Chinese firms have significantly increased their CSR reporting.

China’s CSR practices and disclosure are still in developmental stages and Chinese firms are under greater pressure to involve in CSR-related activities and disclosure (McGuinness et al., 2017). The country’s approach toward CSR is markedly different from that of other countries. CSR is unique in China in that it is endorsed by the Chinese government as the desired activity as a part of its political agenda (Yin and Zhang, 2012) and Chinese firms engage in strategic CSR activities in response to government pressure. Another feature that distinguishes Chinese firms from their western counterparts is their extensive political connections (PCs), most notably the close ties between the government and the firm’s senior management (Tu et al., 2013; Haveman et al., 2017). Even though many firms have transitioned from state-owned enterprises to publicly traded firms, the Chinese government retains control and ownership of these firms as the largest shareholder (Guthrie, 2012). Regulatory pressure from the government may have a significant impact on how politically embedded firms (PEFs) in China behave in terms of CSR. PEFs are the main targets of the Chinese government when it comes to enforcing CSR regulations. Besides, Chinese firms’ CSR reporting behavior has been influenced by a variety of factors including firms’ listing status, ownership structure, and legislative upgrading of the corporate governance system. In addition, board gender diversity (BGD) is another important factor that has recently been studied in relation to CSR reporting. The key motivational factor behind increasing the gender diversity of board members is to promote CSR activities, and the presence of women on a company’s board has a considerable influence on the board’s willingness to seriously consider CSR (Bear et al., 2010). The higher the percentage of women on a company’s board of directors, the better the company’s CSR performance. In many countries, the lack of female directors and increasing global pressure for the nomination of more female directors at the top management level have prompted policymakers and regulators to enact gender quotas, and several countries have implemented female quotas on their corporate boards. China has also extended and reformed its corporate governance laws to meet international institutional requirements; however, such woman board representation quota is minimal in China. Gender diversity on corporate boards is considered as an essential strategic management tool for managing stakeholder expectations, and it has emerged as the most common strategy used by businesses to manage stakeholder expectations, particularly their demand for more transparency as concerns CSR reporting (Sial et al., 2018). Further, in China, PEFs are exposed to greater government pressure and must retain their political legitimacy; therefore, their strategic responses may differ from those of firms without political embeddedness. In this light, it is imperative to inquire about how CSR reporting choices in PEFs are affected by female directors on Chinese corporate boards. Therefore, this study specifically addresses the relationship between BGD and PCs and the strategic CSR choices of Chinese firms. This would enable policymakers to formulate policies measures targeted at the legislation of women quota and existence of PCs on Chinese corporate boards to improve firms’ CSR reporting and assist them in achieving their social and environmental objectives more effectively.

We pursue the following main objectives in this study. First, we aim to analyze the extent to which gender-diverse boards in PEFs affect CSR reporting in China. Second, we examine the moderating impact of PCs on the relationship between BGD and firms’ choices of CSR reporting and assess the degree to which CSR reporting varies across PEFs and non-PEFs in response to gender diversity on boards.

A diverse body of empirical attempts has been made in recent years to inquire about the role of BGD in firms’ CSR reporting (Bear et al., 2010; Fernandez-Feijoo et al., 2014; Adams et al., 2015; Al Fadli et al., 2019). Several studies confirmed that the presence of women on BODs has a significant impact on CSR reporting. For instance, Bear et al. (2010) found a positive correlation between women’s representation on BODs and consideration of CSR-related actions. Their findings indicate that gender equality and inclusion of women directors in supervisory boards can play a strategic role in enabling firms to manage sustainable practices and social responsibilities. Fernandez-Feijoo et al. (2014) found that countries with a higher proportion of women on BODs have a higher level of CSR reporting. Some studies pointed out that stakeholders may view gender-diverse boards as an indicator of a higher level of management accountability and social responsibility, and firms with a higher proportion of female directors are likely to engage more in socially responsible actions and disclosure (Adams et al., 2015; Al Fadli et al., 2019). The underlying reason is that women are more concerned about environmental issues, and their values and skills may have a positive influence on firms’ CSR reporting behavior (Terjesen et al., 2009; Adams et al., 2015).

In the case of China, there have been few attempts to examine the role of BGD in CSR reporting by Chinese firms. For example, Liao et al. (2016) and McGuinness et al. (2017) discovered that female directors increase firms’ willingness to engage in CSR activities. In a recent study, Guping et al. (2020) found that BGD has a positive effect on CSR reporting in Chinese firms. Most of the prior research on BGD has concentrated on the underrepresentation of female directors at various board levels. More particularly, previous studies have examined the influence of BGD and PCs on CSR reporting separately, and little attention has been paid to the extent to which gender-diverse boards in PEFs affect CSR reporting, while the literature on BGD’s impact on CSR reporting in the context of China is relatively scarce. This study, therefore, attempts to combine these two lines of research and to assess in a more comprehensive way the uniqueness of CSR reporting behavior of Chinese PEFs having gender diversity in boardrooms.

We investigate the impact of BGD on the CSR reporting decisions made by PEF boards by using an empirical design of Chinese publicly listed firms from 2010 to 2018. The final sample consists of 10,679 firm-year observations. We used fixed-effect regression to explore the above-mentioned phenomenon. Our analysis yields the following main results. First, we find that BGD in PEFs positively affects the CSR reporting decisions. There is evidence that policymakers should devise policies aimed at increasing women’s representation on board and the presence of PCs on Chinese corporate boards to further improve firms’ CSR reporting. Second, we document that PCs moderate the relationship between BGD and firms’ CSR reporting choices. These findings reveal that, in addition to establishing a female quota, policymakers should consider the political ties of board executives to improve women’s role in CSR reporting. Finally, we discover that gender-diverse boards in PEFs are more likely to engage in CSR reporting than non-PEFs boards, implying that PEFs and non-PEFs should be treated differently when it comes to designing CSR strategies.

This study contributes to the existing literature in four ways. First, given the uniqueness of the Chinese business sector, its specific social and political aspects, and the importance of CSR in the Chinese corporate sector, this study attempts to provide empirical evidence on CSR reporting by Chinese firms with BGD, thereby contributing to the body of knowledge confirming the Chinese perspective on CSR disclosure. Second, this study is the first to examine the distinct effect of BGD on CSR reporting decisions made by PEFs and non-PEFs, allowing us to go a step further and present a more fine-tuned picture of the mechanisms underlying the BGD-CSR relationship by examining the previously unexplored moderating effect of PCs on the BGD-firms’ CSR reporting link. Third, we contribute to contemporary CSR research by using a longitudinal approach that is uncommon in the greater China region (Yin and Quazi, 2018). Finally, by considering the role of political connectedness as a driver of firms’ CSR practices, we complement the strand of literature consisting of non-financial reporting which mostly emphasizes the societal and business causes of corporate social responsibility.

The remainder of the paper is structured as follows: Section Institutional background describes the institutional background, while Section Literature Review, Theoretical Background, and Hypotheses provides a review of literature, discusses theories used to explain a firm’s CSR conduct and develops the research hypotheses. The study design and methodology are given in Section Research Method. Sections Empirical Results and Discussion of Results present the findings and discussion of results, respectively. Section Robustness Tests provides robustness tests to check the validity of our findings and finally, and Section Conclusion concludes the paper.

## Institutional Background

In emerging economies, CSR consideration and implementation, as well as CSR shifts, are a relatively recent phenomenon. China, in particular, is relatively a newcomer to the area of CSR engagement and thus provides an excellent empirical setting for studying firms’ CSR-related strategic actions for a variety of reasons. First, China’s corporate governance system is still insufficiently developed to ensure adequate legal protection to investors, indicating that minority shareholders are largely exposed to expropriations by majority shareholders (Allen et al., 2005). Following the year 2000, numerous regulations have been introduced in response to investors’ demand for transparency and effectiveness in the governance system, including the Code of Corporate Governance (2001) and Disclosure Requirements for Companies (2007). Although China has an official regulator, the China Securities Regulatory Commission (CSRC), the country has been unable to synchronize necessary complementary reforms in areas such as corporate laws, investor protection, and property rights (Ma et al., 2013). To increase the effectiveness and transparency of such imperfect corporate governance systems’ decision-making processes, researchers argued strongly for gender diversity on boards, specifically gender equality and the inclusion of women on supervisory boards (Sial et al., 2018). Consequently, many countries including France, Norway, Spain, Italy, Germany, Belgium, Pakistan, and India have enacted quotas for females’ representation on corporate boards, while China has yet to do so.

Second, despite its transition from a highly centralized system to a modern market-oriented economy over the last few decades, China’s economy has retained a reputation for strong government control (Lee et al., 2014; Xu and Zeng, 2016), and the government is still the largest shareholder and keeps the *de facto* control of these companies, regardless of the privatization of many government entities (Guthrie, 2012). Additionally, the political ties between government and firm’s senior management are still popular in China and many firms have government officials as their executives (Ma and Parish, 2006; Tu et al., 2013). These firms are supposed to get many advantages because of their PCs, such as lower taxation, preferential access to debt financing and government projects, and regulatory protection (Faccio, 2010).

China provides a unique institutional setting for examining how PEFs respond to government signals such as the promotion of sustainable business conducts, particularly CSR. The importance of business activities in terms of environmental and social impacts is evident in China’s economic development in the post-reform era (See, 2009). Furthermore, the Communist party’s statements promoting social responsibility among corporations, citizens, and all kinds of institutions, as well as the Shenzhen and Shanghai stock exchanges issuing guidelines for CSR reporting, demonstrate that the Chinese government views CSR as a desirable action (Geng et al., 2010). Accordingly, we can say that government is the major driving force behind CSR in China. Against the above-mentioned institutional particularities, this study will fill a critical gap in the existing CSR literature by exploring whether PCs in firms with female directors on their boards affect CSR reporting choices, in the context of the world biggest emerging economy China, which is constantly facing rapid social and climate changes with every change posing a new challenge for researchers in the field.

## Literature Review, Theoretical Background, and Hypotheses

### CSR Reporting and BGD

A considerable body of literature has been conducted to describe the relationship between CSR and various board attributes including gender, age, directors’ multiple dictatorships, educational background, and nationality. Besides, few studies have explored firms’ CSR reporting behavior regarding BGD, and findings have been inconsistent across institutional contexts. For example, some studies evidenced a positive association between BGD and CSR reporting (Galbreath, 2011; Hafsi and Turgut, 2013; Harjoto et al., 2015; Yasser et al., 2017; Guping et al., 2020). In contrast, several studies reported a negative relationship (Zahra and Stanton, 1988; Molz, 1995), while others found no evidence in support of the BGD and CSR relationship (Coffey and Wang, 1998; Stanwick and Stanwick, 1998).

Numerous theories have been developed to explain firms’ CSR behavior in terms of both internal and external CSR drivers. Our argument in this paper is based primarily on a synthesis of agency theory (internal driver) and legitimacy theory (external driver), which fits our study context well. According to the CSR perspective of legitimacy theory, CSR contributes to the maintenance of congruence between the objectives of firms and societal objectives. Companies may use a variety of reporting strategies, most notably CSR reporting, to legitimize their activities (Chen et al., 2011; Lanis and Richardson, 2013) and gain social approval from their operating environment. The legitimacy and CSR reporting relationship tends to be stronger in response to a regulation or a policy change influencing the public expectations (Rashid, 2018). Thus, firms may attempt to close the perceived legitimacy gap and use CSR reporting to influence the public perception of its actions and responsibilities. By combining the aforementioned theories, we can gain a better understanding of the extent to which managerial pursuance of personal benefits from CSR activities (agency theory) is enabled or constrained by the external societal context (legitimacy theory).

Apart from other aspects of corporate governance, BGD improves not only the control and monitoring of the firms’ decision-making but also enhances their relationships with stakeholders, including the general public (Ellis and Keys, 2015). According to legitimacy theory, the presence of female directors on board motivates firms to use CSR reporting as a strategy for legitimation (Willows and van der Linde, 2016). This is because female’ representation in boardrooms brings social and ethical issues into board discussions and improves the board decision-making quality, thereby helping firms to manage sustainable practices and social responsibilities in a strategic manner (Sartawi et al., 2014). In addition, women have a greater tendency to expand firms’ CSR initiatives due to better understanding and knowledge of their surroundings than their males (Muttakin et al., 2015). Consequently, firms with gender-diverse boards may enhance their CSR reporting to establish legitimacy through strong corporate governance and greater investor appeal (Chan et al., 2014). From the perspective of agency theory, firms’ CSR disclosure is driven by the conflicting incentives of managers, owners, and other stakeholders due to the separation of ownership and control. As it is well-known, BODs are accountable for safeguarding all stakeholders’ interests against opportunistic behavior of managers which can be done through various governance mechanisms such as effective monitoring and improving disclosure levels (Barako and Brown, 2008).

For this, the board needs to be effective in its actions which depend on a variety of board attributes, including gender diversity (Bassett et al., 2007). Female directors increase board effectiveness because they come from non-traditional backgrounds, possess vast knowledge, and are more capable of serving multiple boards than males (García-Izquierdo et al., 2018). Also, because of their diverse economic and social backgrounds, female directors place a higher premium on social issues and the welfare of all stakeholders than on the welfare of a single group (Wang and Coffey, 1992). Further, gender diversity on boards is often seen as an indication of increased managerial accountability and social obligation (Al Fadli et al., 2019). Hence, agency disputes can be resolved by having more women on boards of directors which assists firms to better recognize and interact with the environment through disclosure of CSR-related activities, eventually facilitating firm-stakeholders relationships (Beckman and Haunschild, 2002). As a result of the expectation that gender-diverse boards will have a beneficial effect on firms’ CSR reporting, we hypothesize the following:

*H1*: Firms with gender-diverse boards are more likely to engage in CSR reporting.

### CSR Reporting and PCs

PEFs are defined as firms with strong government ties, whether through network connections or state ownership. While these firms may benefit from easy access to government resources, they are expected to confront high monitoring and show more adherence to government signals to maintain political legitimacy (Marquis et al., 2011). In a country like China, where the government holds the majority of a firm’s shares and CSR reporting is deemed as a desired activity, firms can gain political legitimacy in such an environment by issuing CSR reports in response to government signals, including numerous CSR reporting guidelines issued by the Chinese central government as a strategy to help firms in balancing China’s massive economic growth with its environmental and social effects to pursue the idea of “harmonious society” (Wang et al., 2018).

Unlike non-connected peers, PEFs may not have enough motives to issue CSR reports as they have inherent political legitimacy (Marquis and Qian, 2014); however, this argument negates a control-oriented perspective, which claims that government regulatory pressure can significantly shape the PEFs’ CSR behavior (Huang and Kung, 2010; Zhao, 2012). This is particularly true in China where targeted firms for implementation of government policies are mostly PEFs (Zeng et al., 2012). As a substantial shareholder and a critical source of legitimacy, the government can directly influence firms’ CSR actions. Alternatively, the government can also exert indirect influence on the CSR activities of firms through politically connected board executives. PEFs tend to invest more in CSR initiatives because of the personal incentive of politically connected top managers. Executives with political ties than their peers without political ties have higher motivations to maintain their personal political legitimacy (Marquis et al., 2011), e.g., acting in compliance with government regulations and policies such as issuing CSR reports can help them to protect their reputation and ensure their political careers (Patten and Trompeter, 2003). These assumptions are also consistent with the findings of Xu and Zeng (2016), who discovered that managers with a reputation for high CSR investment have a higher likelihood of promotion and other political benefits. Because responding appropriately to government signals is a key aspect of gaining legitimacy (Marquis et al., 2011), Chinese firms are continually growing their CSR activities, notably CSR reporting while adhering to government laws.

From an agency-oriented view, this can be explained by proposing that executives may pursue their personal incentives and may act at the cost of owners and other stakeholders in the presence of conflicting interests (Jensen and Meckling, 1976). PCs on corporate boards can help mitigate these conflicts as politically connected executives are likely to obey government policies and instructions on CSR disclosure (Zeng et al., 2012), thus promoting CSR reporting practices and maintaining a balance among all stakeholders’ interests.

Furthermore, the government, as the most significant stakeholder in a business in China, sits at the top of the CSR pyramid and endorses CSR as a desirable practice. The government pressure and signals concerning CSR prompt Chinese firms to respond strategically; nevertheless, a firm’s strategic response to government signals is likely to be influenced by the extent of government monitoring (Wang et al., 2008). Firms with political ties are usually subject to more stringent oversight by the government and other regulatory institutions than those without such ties (Gu et al., 2013). If these firms fail to meet their social obligations, they will inevitably suffer unfavorable reputational consequences (Wang et al., 2018). As a result, PEFs are more likely to comply with government directives to implement CSR policies than non-PEFs who are not subject to the same level of government scrutiny. Following these arguments, we test the following hypothesis.

*H2*: PEFs are more likely than non-connected peers to issue CSR reports.

### Moderating Effect of PCs

Firms may use CSR reporting as a strategy for legitimizing their activities to gain social approval and sustain their reputation. Female representation on BODs is one of the critical corporate governance characteristics that support this legitimation strategy. Females on board support this strategy due to their numerous advantages over their male counterparts. For example, they have a better understanding of market environments and use a variety of visions to aid problem-solving (Campbell and Mínguez-Vera, 2008). Females are more risk-averse and possess a broader range of preferences (Croson and Gneezy, 2009). Additionally, they tend to follow less aggressive strategies and invest more in social sustainability initiatives than in research and development (Apesteguia et al., 2012), demonstrating a higher level of ethical concerns and pro-social actions behavior to help firms achieve greater social sustainability (Galbreath, 2011). As a result, firms with gender-diverse boards are expected to strengthen their CSR reporting to maintain legitimacy.

Further, the corporate environment in China has been affected by distinct social and political elements. To achieve a harmonious society, various government policies and enforcements actions have created uncertainty for firms and limited their operations (Hillman et al., 2002). To overcome these constraints and uncertainties, Chinese firms tend to build PCs to gain legitimacy and increase their access to resources and information (Hillman, 2005). Board executives with PCs are expected to have a vast knowledge of regulations and policies regarding CSR as (Gu et al., 2013) asserted that firms with politically connected senior managers may have a higher level of awareness and adoption of CSR policies. Accordingly, boards with political ties are more likely to push firms to show their commitment to government initiatives by responding to government pressure and policy signals regarding CSR reporting (Marquis and Qian, 2014). For that, they are expected to positively influence firms’ decisions on CSR reporting. It is obvious from the above argumentation that both corporate governance attributes (BGD and PCs) are more concerned about the adoption of CSR activities particularly, issuing CSR reports to gain legitimacy. Also, from an agency theory perspective, women and PCs on boards can help to resolve agency conflicts through CSR disclosure showing care and concern for all stakeholder groups and facilitating relationships with them (Beckman and Haunschild, 2002). Therefore, we expect that PCs on boards may enhance the impact of BGD on firms’ CSR reporting. Accordingly, we test the following hypothesis.

*H3*: PCs on boards moderate the relationship between BGD and CSR reporting such that gender-diverse boards with PCs are more likely to issue CSR reports.

## Research Method

### Sample and Data

The sample for this study was drawn from Hexun’s CSR database over a 9-year time span ranging from 2010 to 2018. We started our sample period in 2010 because Hexun launched the CSR evaluation database of all firms listed on the Shenzhen and Shanghai stock exchanges in the year 2010 (Xiong et al., 2016). Recently, many researchers highlighted the prominence of Hexun in China in guiding the investors’ awareness of content and quality of overall CSR reporting activities of listed firms (Li et al., 2013; Xiong et al., 2016). Moreover, according to the user satisfaction and web hit counts, Hexun ranks top in delivering financial information of publicly traded companies by collaborating with the Shanghai Stock Exchange and Thomson Reuters. Data on political connections of board directors and other financial and nonfinancial information were extracted from the China Stock Market and Accounting Research (CSMAR) database, and individual firm annual reports.

We excluded firms belonging to the financial sector because of their unique regulatory characteristics and non-comparability of financial ratios with other industrial sectors. After excluding firms with missing data, the final sample contains 10,679 firm-year observations.

### Variables and Measurements

#### CSR Reporting

We used two measures of CSR reporting to test the study hypotheses. First, we used Hexun CSR rating (CSR score-CSRS) as a proxy for CSR disclosure. CSR rating offered by the Hexun database has been used as a proxy for CSR disclosure in many prior studies (Xiong et al., 2016; Shi et al., 2018b). Hexun’s original CSR measure, constructed on stakeholder theory, covers all five critical stakeholder groups in its assessment framework including environment, community, employees, suppliers and customers, and shareholders. Each of these dimensions is further sub-divided into multiple sub-dimensions, which are weighted differently depending on the industries to which the firm belongs. Even though earning profits for shareholders is considered as a basic corporate responsibility, Hexun’s CSR measure places greater emphasis on stakeholder interests (Xiong et al., 2016). To align with prior research, we exclude the shareholder dimension from Hexun’ original CSR measurement and evaluate CSR on four dimensions (namely environment, community, employees, and supplier & customer) by a total of 19 sub-dimensions (see Xiong et al., 2016, p. 231 for a complete list of Hexun’s CSR measuring items). Second, we created a CSR dummy variable (CSRD) as an alternative measure of CSR reporting to confirm the validity of our main results.

#### Board Gender Diversity

BGD is gauged using four different metrics. We used the Shannon index-SI (Shannon, 1948) as a comprehensive and superior measure of BGD, following the literature (Sial et al., 2018; Ain et al., 2021). In addition, consistent with previous studies (Liu et al., 2014; Trinh et al., 2020), we measured BGD using the Blau index-BI (Blau, 1977), a female director dummy (FDM), and the percentage of female directors (FDBD) for robustness checks. The Shannon index and Blau index (similar to the two widely used measures of diversification in the area of economics and financial studies, i.e., the entropy index and the Herfindahl–Hirschman index, respectively) are the two composite measures of BGD indicating that whether or not the boards are diverse in term of gender and produce the similar results, but the former are larger than the latter (Abad et al., 2017). Because it is a logarithmic matric, the Shannon index is more sensitive to small changes in BGD.

### Political Connections

The presence of PCs in a company’s board is measured in two ways. First, following (Wang et al., 2008; Chen et al., 2011), a dummy variable (PCD) is used that takes the value of 1 if any of the firm’s senior managers, supervisors or directors was or is a member of the Chinese People’s Political Consultative Conference (CPPCC), a government official, or a representative of National People’s Congress (NPC) and zero otherwise. Second, a continuous variable, the percentage of politically connected directors on the board (PC%), is used to examine the overall degree of political ties on the board.

#### Control Variables

We added some corporate governance factors and firm-specific factors as control variables. Corporate governance factors which may influence firms’ CSR reporting include CEO duality (CEOD), board size (BS), board independence (BID), board age (BA), Big 4, state-owned enterprise (SOE), and board meeting frequency (BMF), while firm-level controls include leverage (LEV), firm size (SZ), return on assets (ROA), firm growth (FG), and free cash flows (FCF). In addition, industry and year dummies are included to control for time and sector effects, respectively. Table 1 shows the details of variables measurement.

**Table 1:** Variable measurement.

Variables	Calculations	Variable source
**Dependent variables**		
CSRS	CSRS is the CSR score computed as the aggregate of four sub-dimensions scores from the Hexun database, namely community, environment, customers and suppliers, and employees.	Xiong et al., 2016; Shi et al., 2018b
CSRD	CSRD is a dummy whose value is 1 if a firm is a CSR report issuer and 0 otherwise.	Cabeza-García et al., 2018; Wang et al., 2018
**Independent Variables**		
SI & BI	Shannon index (SI) and Blau index (BI) are measured as $\overset{n}{\underset{i=1}{âˆ‘}}P_{i}\ln P_{i}$ , and $1âˆ’\overset{n}{\underset{i=1}{âˆ‘}}{P_{i}}^{2}$ , where n represents the number of categories (male and female) and P_i_ indicates the percentage of each category in the board.	Sial et al., 2018; Trinh et al., 2020
FDM	FDM is a dummy variable that is equal to 1 if there is at least one female on the board and 0 otherwise.	Al Fadli et al., 2019; Ain et al., 2021
FDBD	FDBD is the percent of female directors computed as the number of female directors on the board divided by board size.	Hafsi and Turgut, 2013; Yasser et al., 2017; Cabeza-García et al., 2018; Beji et al., 2021
**Moderator**		
PC	PC is a dummy variable that takes the value of 1 if one of the senior managers, supervisors, or directors of the firm were or are the Chinese People’s Political Consultative Conference (CPPCC) member, government official, or representative of the National People’s Congress (NPC) and 0 otherwise.	Wang et al., 2008, 2018
PC%	Percentage of politically connected board directors computed as the number of politically connected directors on the board divided by the total number of board directors	Wang et al., 2008; Shi et al., 2018a
**Controls**		
CEOD	CEOD is a dummy variable equal to 1 If the CEO and Chair of the Board are the same, otherwise 0.	Ma et al., 2013; Yasser et al., 2017; Beji et al., 2021
BS	BS is the board size which indicates the total number of board directors.	Yasser et al., 2017; Sial et al., 2018; Beji et al., 2021
BID	BID is the board independence calculated as the total number of board independent directors divided by board size.	Ma et al., 2013; McGuinness et al., 2017
BA	BA is the board directors’ average age.	Yasser et al., 2017; Sial et al., 2018
Big 4	A dummy variable equal to 1 if the company’s auditor is an audit firm belonging to “big 4” or its joint venture in China and 0 otherwise.	Sial et al., 2018; Gull et al., 2021
SOE	A dummy variable takes a value of 1 if the firm is state-owned and 0 otherwise.	Ain et al., 2021; Gull et al., 2021
BMF	Board meeting frequency is computed as the number of board directors’ meetings each year.	Yasser et al., 2017; Sial et al., 2018
LEV	Leverage is computed as total debts over total assets.	Xu and Zeng, 2016; Al Fadli et al., 2019; Beji et al., 2021
FS	Firm size is calculated by taking the natural logarithm of total assets.	Zhao, 2012; Shi et al., 2018b
ROA	Return on assets is computed as EBIT over total assets.	Ma et al., 2013; Beji et al., 2021
FG	Firm growth is measured as the change in a firm’s total assets.	Agrawal and Chatterjee, 2015; Ain et al., 2021
FCF	Free cash flows are calculated as the subtraction of the sum of capital expenditure and working capital from the sum of net profit, noncash expenses, and interest expenses.	Wang et al., 2008; Marquis and Qian, 2014

### Model Specifications and Estimation Technique

The following multivariate regression models were estimated to test our hypotheses:


$$\begin{array}{c} CSRS_{\mathrm{it}}={\mathrm{Î²}}_{0}+{\mathrm{Î²}}_{1}SI_{\mathrm{it}}+{\mathrm{Î²}}_{2}PC_{\mathrm{it}}+{\mathrm{Î²}}_{3}CEOD_{\mathrm{it}}+\\ {\mathrm{Î²}}_{4}BS_{\mathrm{it}}+{\mathrm{Î²}}_{5}\mathrm{BI}D_{\mathrm{it}}+{\mathrm{Î²}}_{6}BA_{\mathrm{it}}+{\mathrm{Î²}}_{7}BMF_{\mathrm{it}}+\\ {\mathrm{Î²}}_{8}\mathrm{Big}\;4_{\mathrm{it}}+{\mathrm{Î²}}_{9}\mathrm{SO}E_{\mathrm{it}}+{\mathrm{Î²}}_{10}LEV_{\mathrm{it}}+{\mathrm{Î²}}_{11}FS_{\mathrm{it}}+\\ {\mathrm{Î²}}_{12}ROA_{\mathrm{it}}+{\mathrm{Î²}}_{13}FG_{\mathrm{it}}+{\mathrm{Î²}}_{14}FCF_{\mathrm{it}}+{\mathrm{Î²}}_{15}\mathrm{Industry}+\\ {\mathrm{Î²}}_{16}\mathrm{Year}+{\mathrm{Î²}}_{17}\mathrm{Province}+{\mathrm{Î²}}_{18}\mathrm{City}+{\mathrm{Îµ}}_{\mathrm{it}} \end{array}$$ (1)

$$\begin{array}{c} CSRS_{\mathrm{it}}={\mathrm{Î²}}_{0}+{\mathrm{Î²}}_{1}SI_{\mathrm{it}}+{\mathrm{Î²}}_{2}PC_{\mathrm{it}}+{\mathrm{Î²}}_{3}SI_{\mathrm{it}}âˆ—PC_{\mathrm{it}}+\\ {\mathrm{Î²}}_{4}CEOD_{\mathrm{it}}+{\mathrm{Î²}}_{5}BS_{\mathrm{it}}+{\mathrm{Î²}}_{6}\mathrm{BI}D_{\mathrm{it}}+{\mathrm{Î²}}_{7}BA_{\mathrm{it}}+{\mathrm{Î²}}_{8}BMF_{\mathrm{it}}+\\ {\mathrm{Î²}}_{9}\mathrm{Big}\;4_{\mathrm{it}}+{\mathrm{Î²}}_{10}\mathrm{SO}E_{\mathrm{it}}+{\mathrm{Î²}}_{11}LEV_{\mathrm{it}}+{\mathrm{Î²}}_{12}FS_{\mathrm{it}}+{\mathrm{Î²}}_{13}ROA_{\mathrm{it}}\\ +{\mathrm{Î²}}_{14}FG_{\mathrm{it}}+{\mathrm{Î²}}_{15}FCF_{\mathrm{it}}+{\mathrm{Î²}}_{16}\mathrm{Industry}+{\mathrm{Î²}}_{17}\mathrm{Year}+\\ {\mathrm{Î²}}_{18}\mathrm{Province}+{\mathrm{Î²}}_{19}\mathrm{City}+{\mathrm{Îµ}}_{\mathrm{it}} \end{array}$$ (2)

where CSRSit reflects the firm’s selection of CSR activities during the sampled period. The independent variables SIit and PCit explain the variation in CSR reporting behavior of the firms under consideration, based on the existence of gender diversity and political ties in boards, respectively. The interaction term SI_it_*PC_it_ describes the effect of political embeddedness on CSR reporting in firms with gender-diverse boards.

To test our hypotheses, a multivariate regression model of panel data, controlling for year and industry fixed effects with firm-level clustered standard errors is used. We also included dummies for provinces and cities because China’s institutional arrangements vary significantly across provinces and cities. In the longitudinal or panel dataset, it is common to use a fixed-effect model to control for omitted variables; however, we confirmed the choice between random effects and fixed effects models *via* the Hausman test.

## Empirical Results

### Descriptive Statistics

Table 2 reports the descriptive statistics of our test and control variables. The results indicate that approximately 32.1% of firms in the total sample issue CSR reports and have an average CSR score of 35.1%. According to the Shannon index, there are on average 30.5% female directors on the board. Notably, around 46.5% of sampling firms have at least one of the senior managers, supervisors, or directors with political ties, whereas politically connected directors account for approximately 16.2% of the total number of board directors. Around 59.2% of the total sample firms are state-owned. CEO duality exists in more than 20% of sampled firms. The average board size is 8.72 and the average board member age is around 49.16 years. In addition, sampling firms have approximately 37.2% independence in their boards and board directors on average, and tend to have more than 9 meetings each year. The average debt ratio and firm size of sampling firms are 52.6 and 21.78, respectively.

**Table 2:** Descriptive statistics.

Variables	Mean	Std. Dev.	Min	Max
CSRS	0.351	0.145	0.154	0.683
CSRD	0.321	0.467	0.000	1.000
SI	0.305	0.179	0.000	0.857
PC	0.465	0.498	0.000	1.000
PC%	0.162	0.075	0.000	0.666
CEOD	0.209	0.406	0.000	1.000
BS	8.725	1.705	4.000	18.000
BID	0.372	0.040	0.182	0.750
BA	49.165	3.880	25.000	88.000
BMF	9.994	4.078	1.000	20.000
Big 4	0.176	0.381	0.000	1.000
SOE	0.592	0.491	0.000	1.000
LEV	0.526	0.166	0.259	0.775
FS	21.783	1.314	7.955	27.381
ROA	0.045	0.147	−0.024	0.229
FG	0.189	0.532	−1.300	3.309
FCF	0.049	0.017	−0.497	0.412

### Correlation Analysis

The results of correlations analysis are given in Table 3. Overall, results indicate the absence of multicollinearity. The strongest correlation of 47.78% is reported between BID and BS which is well-below the established cut-off of 80% (Gujurati and Porter, 2009). Additionally, the values of all variance inflation factors (VIF) are less than 2 which is well under the maximum limit of 5.3 (Hair et al., 2006). Hence, multicollinearity does not pose any serious threat to our model estimation.

**Table 3:** Correlation matrix.

	CSRS	SI	PC	PC%	CEOD	BS	BID	BA
CSRS	1.0000							
SI	0.0261**	1.0000						
PC	0.0211**	0.0067*	1.0000					
PC%	0.0312**	0.0045**	0.2108**	1.0000				
CEOD	0.0383***	0.0344*	0.0088	0.0034	1.0000			
BS	0.0004**	0.0020	0.0277*	0.014**	0.0072*	1.0000		
BID	0.0144*	0.0130*	0.0320*	0.0231*	0.0033	−0.4778*	1.0000	
BA	0.0167*	0.0243*	0.0036*	0.0015*	0.0001	0.0097**	0.0091*	1.0000
BMF	0.0292*	0.0028***	0.0140**	0.0201**	0.0276*	0.0307*	0.0254*	0.0226*
Big 4	0.0519*	0.0039*	0.0008***	0.0012*	0.0370*	0.0151*	0.0049*	−0.0064*
SOE	−0.0282*	0.0425*	−0.0201*	−0.0316**	0.0269*	0.0221*	0.0059	0.0181*
LEV	0.0252*	0.0215*	0.1676*	0.0531*	0.0270*	0.0031	0.0158	0.0009*
FS	0.0329*	0.0054	0.3165*	0.2161*	0.0068	0.2097*	0.0397*	0.0030**
ROA	0.0041*	0.0052	0.0178*	0.0219**	−0.0061	0.0273*	−0.0001	0.0037*
FG	0.0051**	−0.0058**	0.0279*	0.0154*	−0.0011	0.0405*	0.0032	0.0048*
FCF	0.0288**	0.0191*	0.1247*	0.1372*	−0.0007	0.0611*	0.0336*	−0.0111
	**BMF**	**Big 4**	**SOE**	**LEV**	**FS**	**ROA**	**FG**	**FCF**
BMF	1.0000							
Big 4	0.0144**	1.0000						
SOE	−0.0008**	−0.0323*	1.0000					
LEV	−0.0230*	0.0265*	−0.0048*	1.0000				
FS	0.0189*	−0.0432*	0.0154**	0.2097*	1.0000			
ROA	−0.0126	−0.0026	0.0122*	0.0273*	−0.0400*	1.0000		
FG	0.0000***	0.0014	0.0023	0.0405*	0.1303*	0.0001	1.0000	
FCF	0.0040**	0.0054	−0.0290*	0.0611*	0.4251*	−0.0044	0.1966*	1.0000

*, **, *** Represent the significance levels at *p* < 0.1, *p* < 0.05 and *p* < 0.01, respectively.

### Multivariate Analysis

This section presents the empirical investigation of the impact of gender-diverse and politically connected boards on firms’ CSR reporting practices. Table 4 reports the corresponding results. Model 1 shows the results for the direct effect of BGD on CSR reporting using the Shannon index (SI). Findings indicate that the coefficient of SI (β = 0.031, *p* < 0.05) in Model 1 is statistically significant and positive. These findings support H1 indicating that firms having gender diversity in boards are more likely to involve in CSR reporting and are in total alignment with studies (Hafsi and Turgut, 2013; Yasser et al., 2017; Guping et al., 2020) which also reported a positive association between firms’ CSR reporting and BGD. These results imply that Chinese firms having female directors on boards are likely to disclose more CSR-related information because female directors compared to their male counterparts have different values and concerns regarding disclosure of social responsibility-related practices (Bear et al., 2010). Although female directors have a comparatively low proportion on corporate boards in China; however, their presence can be more effective for firms in managing the public perception of their activities and the legitimation process through enhanced awareness and adoption of CSR reporting.

**Table 4:** Do gender-diverse boards in Chinese firms with PCs affect CSR reporting?

	Direct Model		Indirect Model	
	Model 1 (CSRS)		Model 2 (CSRS)	
Variables	Coeff.	*value of p*	Coeff.	*value of p*
SI	0.031**	0.034		
PC	0.043**	0.021	0.0.023***	0.003
SI*PC			0.219***	0.002
CEOD	0.013***	0.002	0.015**	0.041
BS	0.051**	0.044	0.049*	0.061
BID	0.076*	0.068	0.072*	0.060
BA	0.001*	0.078	0.002*	0.072
BMF	0.002***	0.002	0.003***	0.003
Big 4	0.019***	0.001	0.017***	0.001
SOE	−0.005**	0.031	−0.009**	0.032
LEV	0.018**	0.030	0.016**	0.022
FS	0.031*	0.057	0.027*	0.069
ROA	0.093**	0.021	0.067*	0.091
FG	−0.163**	0.015	−0.198*	0.068
FCF	0.125*	0.091	0.051*	0.078
Constant	0.021***	0.001		
Year & Industry	Yes		Yes	
Province & City	Yes		Yes	
N	10,679		10,679	
R^2^ (%)	27.19		28.69	
F	197.76		193.89	
Prob>F	0.000		0.000	

* Represent the significance levels at *p* < 0.1.

** Represent the significance levels at *p* < 0.05.

*** Represent the significance levels at *p* < 0.01.

Further, the coefficient of PCs (β = 0.043, *p* < 0.05) in Model 1 shows a significant positive association with firms’ CSR reporting activity supporting H2 that firms with politically connected boards are more likely to engage in CSR reporting than firms without such connections. These findings are in line with prior research (Marquis and Qian, 2014; Wang et al., 2018), which also confirmed the positive influence of PCs on CSR reporting. These results imply that government regulatory pressures influence the CSR behavior of PEFs in China (Huang and Kung, 2010). This could be because the Chinese government wants PEFs to be pioneers in implementing CSR policies by adhering to government guidelines on the adoption of CSR-related activities (Zeng et al., 2012), thus promoting CSR as the desired activity. Therefore, PEFs are likely to act under government signals and publish CSR reports to gain political legitimacy.

Finally, Model 2 shows the interactive effect of BGD and PCs on the disclosure of CSR activities. The results show that the coefficient of interaction term SI*PC (β = 0.219, *p* < 0.01) in Model 2 is positive and statistically significant, thereby confirming H3. These findings reveal that politically connected boards enhance the role of BGD in increasing the firm’s likelihood of issuing CSR reports. Alternatively, we can assert that gender diversity in boards is more effective in improving CSR reporting in PEFs. This is because both female directors and politically connected executives have greater concern for social matters, particularly the adoption and disclosure of CSR activities, depending upon distinctive gender traits of female directors and politically connected executives’ knowledge and a better understanding of CSR-related policies and their incentives in adherence to government signals.

Among control variables, state-owned enterprise status (SOE) and firm growth (FG) have a significant negative association with firms’ level of CSR reporting. These findings agree with studies (Sial et al., 2018; Guping et al., 2020). All other control variables including CEO duality (CEOD), board size (BS), board independence (BID), board age (BA), board meeting frequency (BMF), Big 4, leverage (LEV), firm size (FS), return on assets (ROA), and free cash flows (FCF) are significantly positively related to CSR reporting level. These findings are in alignment with studies (Yasser et al., 2017; Al Fadli et al., 2019).

## Discussion of Results

Our research adds to the current body of knowledge by examining how gender diverse, as well as politically linked corporate boards, impact Chinese listed firms’ CSR reporting practices. We used China’s distinct characteristics to gain a deeper understanding of government signaling and how a company board’s attributes specifically gender diversity in boards impact its reaction to signaling. The study’s findings reveal that the presence of both female directors and politically connected executives on corporate boards improves the firms’ CSR reporting. The results also indicate that PEFs have a higher chance of engaging in CSR reporting than non-PEFs. Furthermore, it has been discovered that having PCs on boards might enhance the influence of BGD on companies’ CSR reporting.

From a theoretical standpoint, our results are in alignment with the legitimacy and agency theories’ perspectives. From a legitimacy theory perspective, businesses must legitimate their activities for survival and growth in the environment and society in which they operate. Conflicts may arise when firms’ goals negate the social and political goals. The CSR perspective of legitimacy theory helps in resolving these conflicts, and firms’ approach toward controlling and resolving these conflicts is an attractive research subject. The Chinese government has been actively signaling to businesses that CSR is a legitimate and vital activity. However, in the corporate environment of China, firms may find it difficult to understand and respond to government directions due to underdeveloped institutional infrastructure and lax enforcement of current standards (Marquis et al., 2011). In such scenarios, we believe that responding to government signals and establishing legitimacy with governmental players is critical. The legitimate status is said to be an absolute necessity for easier access to resources and markets, and long-term survival (Brown, 1998). Furthermore, because the Chinese government, as a major stakeholder in many firms, has control over critical resources that shape their competitive environments and positions, firms are strategic in how they manage their interactions with government entities to strengthen their positions (Hillman, 2005). Research has shown that the more the government’s influence in a firm’s immediate surroundings, the more likely the firm is to participate in political strategies (Bonardi et al., 2005). We found that firms can seek preferred status (i.e., legitimacy) and associated resources from the government by strategically reporting on their social responsibility which implies that Chinese companies are more inclined to pursue CSR reporting as a political strategy.

Furthermore, to safeguard the interests of stakeholders, corporate boards regulate and monitor the decision-making process in firms (Barako and Brown, 2008). For this, boards must be effective which largely depends on gender quota for females on these boards (Bassett et al., 2007). It is argued that because of their strong relation-building quality, females have a greater ability to engage and respond to multiple stakeholders considering it as social responsibility (Galbreath, 2011). Female directors tend to improve the board’s efficiency in terms of environmental policy because they place a greater emphasis on green issues. As per social identification theory and social categorization theory, women directors are more involved in social responsibility initiatives than male directors because they are more concerned about alleged environmental and health risks (Bonardi et al., 2005). The inclusion of female directors on corporate boards increases the use of CSR reporting as a legitimization tool, aiding in the alignment of business and society goals through the mechanism of increasing CSR reporting levels.

Alternatively, the CSR perspective of agency theory can aid in the resolution of interest misalignment in the separation of control and ownership. Female directors on boards guarantee that the firm’s goals and the social effect of its operations are met, as well as for settling agency conflicts and legitimizing the firm’s actions (Guping et al., 2020). This is because they have a higher level of social concern, a better grasp of the firm’s internal and external environment, and the capacity to respond to community-related challenges through CSR disclosure (Beckman and Haunschild, 2002). Voluntary disclosures, such as CSR reporting, help to reduce information asymmetry, allowing the reporting organization to maintain and improve its reputation. As a result, the level of CSR disclosure is an indicator of an efficient and effective board, because it can bring managers’, shareholders’, and other stakeholders’ interests together (Al Fadli et al., 2019).

Our findings also align with the agency-centered perspective of political legitimacy, implying that government-induced CSR policies can be implemented more effectively in the presence of politically connected executives on boards. Since, the government can shape firms’ CSR activities in both a direct and indirect manner by using its majority shareholding status and appointing the board executives with PCs, respectively (Wang et al., 2018). Therefore, in addition to government ownership, PCs can be used as a tool for the diffusion of CSR-related practices in China. We observed that indirect government pressure in the form of political embeddedness is a strong predictor of CSR reporting in our developing market environment, i.e., a country where the government owns a significant portion of many firms. The board of directors’ membership in government and/or political councils, such as the NPC or CPPCC, affects the company’s legitimacy and, as a result, the likelihood of publishing a CSR report. This, we believe, is because such connections expose the firm to additional scrutiny. Even though research has demonstrated that they are crucial for resource access in China (Wang et al., 2018), this may come at the cost of higher government monitoring. Our results also indicate that when the boards are politically embedded, female directors on boards have a higher effect on firms’ CSR reporting, highlighting that PCs can add more potential to the role of female directors on board to further promote CSR reporting. The findings that PCs have a moderating effect on the female directors’ role in CSR reporting suggest that responding to government signals is not a straightforward process. Firms may experience varying degrees of legitimacy pressure depending on the characteristics of their board executives.

## Robustness Tests

In this section, some robustness tests were conducted to check the sensitivity of our main findings from different perspectives including the alternative measurements of dependent and independent, and moderator variables and controlling for possible endogeneity problems.

### Alternative Measure of CSR Reporting

Table 5 shows the results for the association between BGD and CSR reporting using CSR dummy (CSRD) as a proxy for a firm’s CSR reporting. Findings demonstrate that the coefficients for direct influence of BGD on firms’ CSR reporting [SI (β =0.245, *p* < 0.05)] in Model 1 along with interaction term [SI*PC (β =0.929, *p* < 0.01)] in Model 2 remained significantly positive, showing that our main results, reported in Table 4, persist and remained insensitive to the alternative measurement of CSR reporting.

**Table 5:** Alternative measure of CSR reporting.

	Direct Model		Indirect Model	
	Model 1 (CSRD)		Model 2 (CSRD)	
Variables	Coeff.	*value of p*	Coeff.	*value of p*
SI	0.245**	0.035		
PC	0.078***	0.006	0.298***	0.006
SI*PC			0.929***	0.006
CEOD	0.240***	0.000	0.239***	0.000
BS	0.017**	0.029	0.020**	0.014
BID	0.423*	0.057	0.426**	0.031
BA	0.006*	0.064	0.003**	0.014
BMF	0.039***	0.000	0.032***	0.000
Big 4	0.367***	0.000	0.368***	0.000
SOE	−0.062*	0.072	−0.085**	0.013
LEV	0.307*	0.060	0.303*	0.061
FS	0.034**	0.013	0.027***	0.006
ROA	0.013**	0.018	0.015*	0.051
FG	0.188	0.331	0.187	0.597
FCF	0.179**	0.050	0.202*	0.068
Year & Industry	Yes		Yes	
Province & City	Yes		Yes	
N	10,679		10,679	
Pseudo R^2^ (%)	20.82		21.88	
Wald χ^2^	137.77		147.99	
Prob> χ^2^	0.000		0.000	
Specification test-linktest (Hatsq)	0.463		0.829	
Gof test group (10)	12.05		7.04	
Prob.	0.149		0.532	
% of correction prediction	80.12		80.13	

* Represent the significance levels at *p* < 0.1.

** Represent the significance levels at *p* < 0.05.

*** Represent the significance levels at *p* < 0.01.

### Alternative Measures of BGD

We used Blau index (BI), female director dummy (FDM), and percent female directors (FDBD) as proxies for alternative measurement of BGD. The corresponding results are given in Table 6 which show that in Models 1, 2, and 3, the coefficients of BI (β = 0.131, *p* < 0.05), FDM (β = 0.005, *p* < 0.01) and FDBD (β = 0.021, *p* < 0.01) are significant and positive. Likewise, in Models 4, 5 and 6, the interaction terms BI*PC (β = 0.178, *p* < 0.05), FDM*PC (β = 0.032, *p* < 0.01) and FDBD*PC (β = 0.049, *p* < 0.05) are also found to have a significant positive effect on CSR reporting. These results confirm the main findings given in Table 4. Thus, it is established that our results remain insensitive to alternative measurements of BGD.

**Table 6:** Alternative measures of BGD.

	Direct Models						Indirect Models					
	Model 1 (CSRS)		Model 2 (CSRS)		Model 3 (CSRS)		Model 4 (CSRS)		Model 5 (CSRS)		Model 6 (CSRS)	
Variables	Coeff.	*value of p*	Coeff.	*value of p*	Coeff.	*value of p*	Coeff.	*value of p*	Coeff.	*value of p*	Coeff.	*value of p*
BI	0.131**	0.010										
FDM			0.005***	0.004								
FDBD					0.021***	0.003						
PC	0.046**	0.023	0.003**	0.011	0.002**	0.012	0.021***	0.001	0.005**	0.024	0.007**	0.019
BI*PC							0.178**	0.033				
FDM*PC									0.032***	0.008		
FDBD*PC											0.049**	0.027
CEOD	0.014**	0.049	0.011***	0.002	0.015***	0.001	0.013***	0.003	0.011**	0.023	0.020**	0.029
BS	0.060***	0.003	0.071**	0.037	0.051**	0.048	0.074**	0.026	0.058**	0.021	0.056**	0.019
BID	0.072**	0.032	0.069**	0.029	0.078*	0.056	0.059**	0.017	0.073**	0.015	0.067**	0.021
BA	0.002**	0.024	0.001**	0.014	0.002*	0.083	0.003**	0.019	0.004**	0.011	0.003**	0.011
BMF	0.002**	0.025	0.003***	0.023	0.003***	0.003	0.002**	0.011	0.001*	0.063	0.003**	0.047
Big 4	0.018***	0.001	0.017***	0.000	0.018***	0.001	0.019***	0.000	0.020***	0.007	0.022***	0.005
SOE	−0.007**	0.013	−0.005***	0.007	−0.006**	0.014	−0.006**	0.017	−0.008**	0.019	−0.005**	0.018
LEV	0.021**	0.014	0.019**	0.021	0.018*	0.059	0.017**	0.020	0.019**	0.027	0.017**	0.023
FS	0.001**	0.020	0.001***	0.004	0.004*	0.061	0.002***	0.005	0.003**	0.019	0.003**	0.025
ROA	0.091**	0.034	0.031**	0.040	0.030**	0.017	0.048**	0.019	0.051**	0.021	0.049*	0.069
FG	−0.139*	0.060	−0.081*	0.087	−0.049*	0.071	−0.176*	0.090	−0.201*	0.093	−0.091*	0.091
FCF	0.031*	0.079	0.028**	0.029	0.027**	0.021	0.034*	0.088	0.036**	0.048	0.067*	0.061
Constant	0.056**	0.015	0.051**	0.013	0.039**	0.016	0.053**	0.020	0.061**	0.019	0.049**	0.022
Year & Industry	Yes		Yes		Yes		Yes		Yes		Yes	
Province & City	Yes		Yes		Yes		Yes		Yes		Yes	
N	10,679		10,679		10,679		10,679		10,679		10,679	
R^2^ (%)	31.22		32.09		31.97		33.18		33.27		32.92	
F	187.34		203.10		197.56		206.19		199.34		176.90	
Prob>F	0.000		0.000		0.000		0.000		0.000		0.000	

* Represent the significance levels at *p* < 0.1.

** Represent the significance levels at *p* < 0.05.

*** Represent the significance levels at *p* < 0.01.

### Alternative Measure of PC

To check the reliability of our definition of PC, we re-investigated the CSR reporting choices of firms in association with BGD and PC using the percentage of politically connected directors in the board to the total number of board directors (PC%) as an alternative measure of PC. The results, provided in Table 7, reveal that the coefficients for both the direct and indirect influence, i.e., SI (β = 0.019, *p* < 0.05), PC (β = 0.045, *p* < 0.05; β = 0.012, *p* < 0.01) and SI*PC % (β = 0.275, *p* < 0.01) in Models 1 and 2, respectively, retain the sign and significance and are consistent with those reported in Table 4 using PC dummy. Hence, our results continued to be indifferent to alternative PCs’ measurement.

**Table 7:** Alternative measure of PC.

	Direct Model		Indirect Model	
	Model 1 (CSRS)		Model 2 (CSRS)	
Variables	Coeff.	*value of p*	Coeff.	*value of p*
SI	0.019**	0.015		
PC %	0.045**	0.014	0.012***	0.001
SI*PC %			0.275***	0.000
CEOD	0.011***	0.001	0.019**	0.017
BS	0.007**	0.040	0.009*	0.090
BID	0.076*	0.054	0.056*	0.067
BA	0.002*	0.096	0.003*	0.087
BMF	0.001***	0.003	0.002***	0.000
Big 4	0.019***	0.000	0.021***	0.000
SOE	−0.006**	0.022	−0.005**	0.032
LEV	0.013**	0.012	0.017**	0.043
FS	0.031**	0.010	0.041*	0.079
ROA	0.094**	0.063	0.077*	0.081
FG	−0.165**	0.083	−0.173*	0.091
FCF	0.107*	0.061	0.096**	0.049
Constant	0.018**	0.047		
Year & Industry	Yes		Yes	
Province & City	Yes		Yes	
N	10,679		10,679	
R^2^ (%)	28.00		30.22	
F	154.27		161.11	
Prob>F	0.000		0.000	

* Represent the significance levels at *p* < 0.1.

** Represent the significance levels at *p* < 0.05.

*** Represent the significance levels at *p* < 0.01.

### Control for Endogeneity

In accounting research, endogeneity is a common problem that could arise due to omitted variables, explanatory variables, and other instantaneous consequences. It is suggested that the relationship between board members and CSR reporting can be simultaneous (Velte, 2017). To resolve the possible endogeneity problem, we re-estimated the main model using the generalized methods of moment (GMM) model. The results of the GMM model presented in Table 8 show that the *p*- values of the Sargan test, Hansen test, and AR 2 are all insignificant. Additionally, the signs and coefficients are also like those of the main models. Hence, our results are insensitive to the endogeneity problem. Further, to deal with the problem of reverse causality, we used the lagged values of explanatory variables following the literature (Sial et al., 2018; Ain et al., 2021). Accordingly, we used the lagged values of the BGD measure to re-investigate our research hypotheses. The results, reported in Table 9, confirm the robustness of the main findings in Table 4 thus indicating that reverse causality is unlikely.

**Table 8:** GMM regression.

	Direct Model		Indirect Model	
	Model 1 (CSRS)		Model 2 (CSRS)	
Variables	Coeff.	*value of p*	Coeff.	*value of p*
L1	0.460***	0.000	0.472***	0.000
SI	0.018***	0.000		
PC	0.004***	0.000	0.09***	0.001
SI*PC			0.058***	0.000
CEOD	0.014***	0.000	0.017***	0.000
BS	0.050***	0.000	0.035**	0.040
BID	0.038*	0.070	0.026**	0.036
BA	0.002***	0.000	0.003***	0.000
BMF	0.007***	0.002	0.006***	0.000
Big 4	0.051*	0.056	0.061***	0.001
SOE	−0.010***	0.001	−0.011***	0.000
LEV	0.016***	0.000	0.016***	0.000
FS	0.003***	0.000	0.004***	0.006
ROA	0.045***	0.000	0.064***	0.000
FG	0.054***	0.000	0.072***	0.003
FCF	0.060**	0.020	0.081*	0.082
Year & Industry	Yes		Yes	
Province & City	Yes		Yes	
N	10,679		10,679	
Wald χ^2^	1986.72		1147.99	
Prob> χ^2^	0.000		0.000	
Arellano-Bond test for AR(2)	0.210		0.310	
Sargan test	0.187		0.480	
Hansen test	0.517		0.618	

* Represent the significance levels at *p* < 0.1.

** Represent the significance levels at p < 0.05.

*** Represent the significance levels at *p* < 0.01.

**Table 9:** Reverse causality.

	Direct Model		Indirect Model	
	Model 1 (CSRS)		Model 2 (CSRS)	
Variables	Coeff.	*value of p*	Coeff.	*value of p*
SI	0.011**	0.013		
PC	0.055***	0.003	0.051***	0.007
SI*PC			0.017**	0.022
CEOD	0.012***	0.000	0.003**	0.045
BS	0.009**	0.030	0.004*	0.068
BID	0.070*	0.080	0.016*	0.069
BA	0.003*	0.089	0.001*	0.094
BMF	0.001***	0.000	0.003**	0.026
Big 4	0.014***	0.000	0.010**	0.014
SOE	−0.008***	0.003	−0.007**	0.044
LEV	0.012**	0.013	0.004**	0.017
FS	0.002**	0.030	0.003**	0.012
ROA	0.662**	0.047	0.029*	0.097
FG	−0.876**	0.027	0.137*	0.061
FCF	0.123*	0.062	0.150**	0.024
Constant	0.014**	0.044	0.030**	0.035
Year & Industry	Yes		Yes	
Province & City	Yes		Yes	
N	10,679		10,679	
R^2^ (%)	32.10		35.19	
F	160.02		164.10	
Prob>F	0.000		0.000	

* Represent the significance levels at *p* < 0.1.

** Represent the significance levels at *p* < 0.05.

*** Represent the significance levels at *p* < 0.01.

Finally, we employed the propensity score matching technique (PSM) to address the issue of selective bias. Another possible problem with the validity of our main findings is self-selection bias. This suggests that gender diversified boards have distinct characteristics than non-diversified boards and that it is likely that these characteristics, rather than the presence of female directors on the board, cause firms to engage in CSR reporting. We used PSM and followed the literature (Liu, 2018; Ain et al., 2021) to tackle this problem. First, the logit model was used to predict the likelihood that the firm will appoint female directors, with the same control variables as in the main analysis. For each firm in the treatment group (i.e., firms with female directors), a control group (i.e., firms without female directors) was identified using this procedure. Except for gender diversity, the control group was assumed to have no differentiating characteristics. Table 10 shows the findings of the PSM, providing additional support for our findings.

**Table 10:** Propensity score matching.

	Direct Model		Indirect Model	
	Model 1 (CSRS)		Model 2 (CSRS)	
Variables	Coeff.	*value of p*	Coeff.	*value of p*
SI	0.098**	0.015		
PC	0.179***	0.000	0.242***	0.008
SI*PC			0.623***	0.003
CEOD	0.035**	0.023	0.552**	0.036
BS	0.010**	0.019	0.447***	0.007
BID	0.025*	0.092	0.306**	0.037
BA	0.006*	0.069	0.043**	0.031
BMF	0.004**	0.045	0.151**	0.011
Big 4	0.201***	0.008	0.511***	0.001
SOE	−0.052*	0.062	−0.058**	0.031
LEV	0.108***	0.000	0.068*	0.078
FS	0.105***	0.000	0.263***	0.042
ROA	0.020*	0.086	0.069**	0.021
FG	0.595	0.357	0.232	0.793
FCF	0.384**	0.027	0.761***	0.003
Year & Industry	Yes		Yes	
Province & City	Yes		Yes	
N	10,679		10,679	
Pseudo R^2^ (%)	23.30		22.88	
F	345.31		324.42	
Prob>F	0.000		0.000	

* Represent the significance levels at *p* < 0.1.

** Represent the significance levels at *p* < 0.05.

*** Represent the significance levels at *p* < 0.01.

## Conclusion

In conclusion, this study contributes novel insights and presents reliable evidence that the presence of female directors and political connections on the board are positively associated with a firm’s CSR reporting by controlling various corporate governance and other firm-related factors. We found that female board directors in China tend to raise awareness of CSR reporting practices and Chinese firms with gender-diverse boards have a greater potential to engage in CSR reporting to fulfill their social responsibility and maintain legitimacy. The underlying reason is that female directors have some unique qualities, such as a stronger emphasis on ethical and social concerns, deeper comprehension of and awareness of their environment, and the capacity to respond appropriately to their environment. Further, PCs on company boards improve CSR reporting, therefore companies with political ties are more likely to provide CSR reports than companies without them. This might be because the government wants these firms to be the first to implement CSR policies. Furthermore, politically connected executives may have personal motivations for being politically connected, such as career progression and reputation. Finally, PCs on corporate boards are found to positively moderate the role of female directors in boosting a firm’s CSR reporting, indicating the relevance of PCs in addition to gender diversity in addressing the diffusion of CSR practices in the Chinese corporate environment. We believe that our findings lead to a deeper understanding of the link between gender diversity and CSR reporting, and call for more attention to its impact on CSR.

### Practical and Theoretical Implications

Our findings have several practical and theoretical implications. The findings support the notion that female presence and political links improve the board’s efficacy. Regarding practical implications, these results are crucial for academia, company boards, policymakers, business partners, and investors of Chinese firms to evaluate the impact of board gender diversity and political connections on CSR reporting. First, in the context of academia, investigating this link will contribute to a better understanding of the effects of gender diversity and PCs on CSR reporting. Second, for boards, the study of gender diversity and PCs would benefit in making wiser decisions and improving their companies’ performance, particularly in terms of CSR protection. Gender diversity on boards has a significant positive impact since more female directors can improve critical board processes such as analysis and decision making. This favorable influence of females on boards can improve CSR ratings, which can increase company reputation and have a positive impact on institutional investment, stock price, and financial performance of the firm (Bear et al., 2010). This study gives investors an additional tool to use when evaluating potential investments. Because having more women on a board can improve CSR, board changes can send significant signals to investors about a company’s potential for better reputation and performance.

The study finds robust evidence that women directors can perform a strategic role in assisting firms in managing sustainable practices and social responsibilities ethically. Given that unethical behavior is more common in emerging economies, the policymakers and regulators are recommended to further improve and legislate gender quotas for women on Chinese corporate boards, as they are in other Asian and European countries, to further improve firms’ internal corporate governance mechanism and to aid in the more achievement of social and environmental goals. However, in the current business environment, improving females’ board representation is a lengthy process fraught with obstacles including dismissal from informal networks, unfriendly corporate culture, and male stereotyping (Ragins et al., 1998). As a result, policymakers must undertake some professional training to develop skills and create a reasonable competitive environment for females to stimulate their career development.

Further, results support the argument that PCs can be a significant driver of a firm’s CSR activities and that PEFs reports differently on CSR than non-PEFs implying that government should implement different policies for PEFs and non-PEFs to exert a stronger influence on their strategic CSR choices. Moreover, the findings show that PCs assist female directors in boosting enterprises’ CSR reporting, implying that in addition to establishing women quotas on boards, political ties of board executives should also be encouraged to further promote CSR in China’s corporate environment. Finally, investors and business partners interested in improving Chinese firms’ corporate social performance should encourage the appointments of senior executives with political ties and appreciate an institutional environment suitable for CSR initiation.

In terms of theoretical implications, our study contributes to agency theory and legitimacy theory by demonstrating that having more female directors and PCs on the board improves the board’s performance and firm’s legitimacy regarding social responsibility, notably CSR reporting. Agency theory relates board gender diversity with a firm’s disclosure of CSR activities and claims that agents disclose such information as a result of the incentives they can gain from such disclosure activities such as reduction in information asymmetry and settlement of agency disputes (Barako and Brown, 2008; Li et al., 2018). The legitimacy theory, on the other hand, link such disclosures to companies’ efforts to justify their conduct in front of shareholders and other stakeholders and female directors promote CSR reporting as a legitimization tactic (Chan et al., 2014; Willows and van der Linde, 2016). Our findings lend support to these theories and extend further by presenting the novel evidence of a positive intervening effect of PCs on boardroom gender diversity in terms of a firm’s social responsibility reporting. We state that female directors have a stronger influence on board decision making and corporate legitimacy as regards CSR when corporate boards are politically active.

Unlike previous research (Chan et al., 2014; Yasser et al., 2017; Al Fadli et al., 2019; Guping et al., 2020) that has focused solely on the influence of female directors in CSR disclosure, our study provides new insights into how country-specific institutional elements such as political connections influence the governance role of female directors in firm’s CSR-related activities and support the recommendations of the world’s regulatory authorities on gender diversity in the boardroom. More specifically, we document that gender diversity on the board has the potential to improve China’s inadequate governance structure and the board’s political links amplify this potential. This will further contribute to a better understanding of the legitimization process for Chinese corporations in terms of CSR reporting, which is highly reliant on the characteristics of board leaders.

### Limitations and Future Research

There are some caveats in this study, which may offer useful insights for future research. First, our research is context-specific, focusing solely on Chinese firms that operate in a distinct social, political, and business environment. Therefore, we urge that future research investigate our findings in other settings, such as developed economies or a sample of different emerging economies together, to see if they are generalizable. Second, because of data limitations, we were unable to add other board directors’ attributes, such as their educational background and qualification level. As a result, future research may investigate their impact on companies’ CSR disclosure practices. Finally, the use of archival data is another drawback in our study, as we are unable to validate how female directors behave in the boardroom when it comes to CSR-related issues. This can be addressed in future research by using primary data such as conducting director surveys and interviews.

## Data Availability Statement

The data used in this study are available on request from the corresponding author.

## Author Contributions

RS and HY contributed to the conceptualization, methodology, investigation and writing - original draft. RS and HB performed the data collection, data curation and formal analysis. RS, MB, and FN participated in the manuscript revision, review, editing and validation. All authors have read and approved the final manuscript.

## Conflict of Interest

The authors declare that the research was conducted in the absence of any commercial or financial relationships that could be construed as a potential conflict of interest.

## Publisher’s Note

All claims expressed in this article are solely those of the authors and do not necessarily represent those of their affiliated organizations, or those of the publisher, the editors and the reviewers. Any product that may be evaluated in this article, or claim that may be made by its manufacturer, is not guaranteed or endorsed by the publisher.
